# The Intercalation of CORM-2 with Pharmaceutical Clay Montmorillonite (MMT) Aids for Therapeutic Carbon Monoxide Release

**DOI:** 10.3390/ijms20143453

**Published:** 2019-07-14

**Authors:** Muhammad Faizan, Kifayat Ullah Khan Niazi, Niaz Muhammad, Yongxia Hu, Yanyan Wang, Dezhi Lin, Yuanyuan Liu, Weiqiang Zhang, Ziwei Gao

**Affiliations:** 1 Key Laboratory of Applied Surface and Colloid Chemistry MOE, School of Chemistry and Chemical Engineering, Shaanxi Normal University, Xi’an 710062, China; 2 School of Materials Science and Engineering, Xi’an Jiaotong University, Xi’an 710049, China; 3 Department of Biochemistry, College of Life Sciences, Shaanxi Normal University, Xi’an 710062, China

**Keywords:** CO, CO-releasing materials, CO-releasing molecules, pharmaceutical clay, montmorillonite, CO kinetic profile, myoglobin assay, therapeutic applications

## Abstract

The pharmaceutical clay montmorillonite (MMT) is, for the first time, explored as a carbon monoxide-releasing material (CORMat). MMT consists of silicate double layered structure; its exfoliation feature intercalate the CORM-2 [RuCl(μ-Cl)(CO)_3_]_2_ inside the layers to suppress the toxicity of organometallic segment. The infrared spectroscopy (IR) confirmed the existence of ruthenium coordinated carbonyl ligand in MMT layers. The energy-dispersive X-ray spectroscopy (EDX) analysis showed that ruthenium element in this material was about 5%. The scanning electron microscopy (SEM) and transmission electron microscope (TEM) images showed that the layer-structure of MMT has been maintained after loading the ruthenium carbonyl segment. Moreover, the layers have been stretched out, which was confirmed by X-ray diffraction (XRD) analysis. Thermogravimetric (TG) curves with huge weight loss around 100–200 °C were attributed to the CO hot-release of ruthenium carbonyl as well as the loss of the adsorbed solvent molecules and the water molecules between the layers. The CO-liberating properties have been assessed through myoglobin assay. The horse myoglobin test showed that the material could be hydrolyzed to slowly release carbon monoxide in physiological environments. The half-life of CO release was much longer than that of CORM-3, and it has an excellent environmental tolerance and slow release effect.

## 1. Introduction

Carbon Monoxide (CO) has a notorious reputation for being silent killer and life-threatening element for living organisms because of its colorless, odorless, and poisonous nature [1]. Using haemoglobin dissociation parameters, Haldane and Douglas, for the first time, scientifically studied that CO poisoning nature is exerted in the carboxy haemoglobin (COHb) [2,3]. This discovery, that revealed the beneficial activity of CO in the living organism as an endogenous gaseous messenger, has attracted greater attention by the researchers to design and develop such mechanisms and strategies that could deliver the reserved CO to the specific tissues at a controlled and moderate rate such as vascular modulator [4,5,6]. Endogenously released gaseous messengers, or gas transmitters, from these particular molecules have been calibrated as nanomedicines (NMs) or nanomaterials (NMs), which are essential for the physiology of all microorganism postulates while performing intra-cellular and inter-cellular approaches [7]. Apart from CO therapy, other gases like nitric oxide (NO) and hydrogen sulphide (H_2_S) are in the pipeline for clinical development. However, few of them are being used as clinical agents or either in-clinical development phase.

The technological innovation has become operational to provide CO for pre-clinical [8] and clinical services (ClinicalTrials.gov identifier: NCT01050712) by means of either inhaled gaseous therapy [9] or potentially parenteral and orally vigorous CO-administration, which has been developed from substantial professional medical chemistry initiative [10]. However, few of them are testified in clinical trials and preclinical models [11,12]. This foundation boosts the awareness of therapeutics gases as regulators of cellular operations. The exogenous CO facility avoiding the CO induction to mainstream blood circulation increases the COHb serum level; consequently, CO became a lethal gas. For CO therapy, it is necessary to engage it with biological stems and become the part of intracellular mechanism. The myoglobin assay famous as “gold standard” can be performed for observing the CO behavior in vitro.

The exogenous endeavor CO-releasing molecules (CORMs) and CO-releasing materials (CORMats) are the appropriate choice for accomplishing the therapeutic activities of CO. The disintegration of CORM/CORMats into CO can be initiated through peculiar activators/conditions and may also apply to control administration for CO migration; such morphology has already been experienced in Photo-CORMs strategies [13,14]. CORMs/CORMats are basically solid forms of CO carriers that require some formulation for releasing the specific amount. That is why we wish to release the CO from its origin—a parent element requiring some administration to lose its grip for holding CO. The toxicity and lacking selectivity of tissue cells of CORMs derived from transition metal carbonyl complex are challenges to develop the clinical agent. To address this issue, developers are shifting from CORMs to CORMats (Table 1).

Several Scaffold/conjugate formulations have been introduced, and are still under investigation to employ compatible conjugate CORMats (Ru-, Mn-, Fe-, metal carbonyl complexes) through various nano-transporting services such as micellar system [15,16,17], peptide [18,19,20,21,22,23], vitamins [24,25,26,27], polymer [28,29], protein [30,31,32,33,34], iron MOFs [35], nanofiber gel [21], inorganic hybrid scaffolds [36,37,38,39], and metallodendrimers [40]. Furthermore, the metal residue after CO releasing inevitably accumulates and dissociated non-carbonyl ligand fragments are probably involved in unpredicted biological toxicity. This metal residue can be managed through an appropriate framework. The development of the solid CO precursor in tandem with peculiar trigger for releasing the enclosed CO gas commodity is an imperative research motive.

Clay minerals are widely used as excipients and active agents in pharmaceutical products. In recent years, clay has been proposed as a very useful material for modulating the drug delivery based on its high retention capacity as well as better swelling and colloidal properties. Clay minerals are naturally occurring inorganic cation exchangers. So, it might be involved in ion exchange reactions with the essential drugs in solution and can be continuously biodegraded by the body organism, especially montmorillonite (MMT) and saponite.

**Figure 1:**
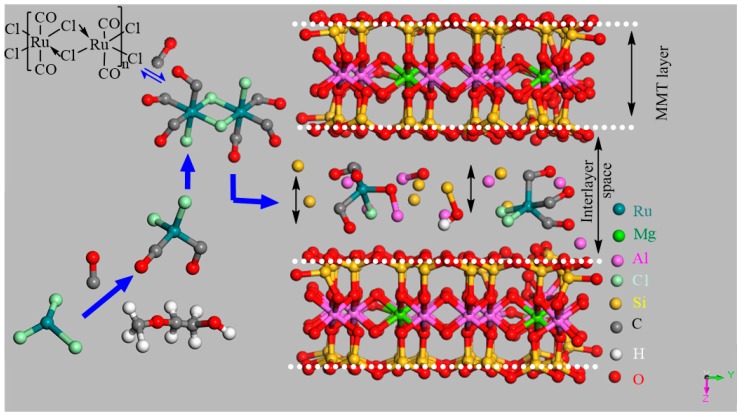
The intercalation of CO-releasing molecule-2 (CORM-2) [Ru(CO)_3_Cl_2_]_2_ inside the Montmorillonite (MMT) layers presented through Material Studio software.

While discussing the montmorillonite intercalation exclusive features, the exfoliation of montmorillonite layers is one of the prime aspects in understanding the ongoing mechanism in it [41]. This exfoliation is usually facilitated by different solvent interactions i.e., poly(ethylene glycol) because of cation exchange capacity (CEC) [42,43]. The as-synthesized morphology has already been thoroughly investigated by different instrumental techniques and computer simulations [44,45]. For computer simulation, *Material studio* is one of the interesting tools to elaborate it (Figure 1) [46]. Our research team envisioned the CEC analogy while paying attention to reducing the toxic nature [47]. MMT is a biocompatible substance and widely used for many applications such as pharmaceuticals [48,49], medicines, drugs [50,51], organo-clay [52,53] and nanoclay [54,55].

The construction of new CORMats is highly dependent on the characteristics of the precursor materials. MMT has a strong capability to absorb the metal carbonyl complexes (MCCs). The nanoporous and nanostructured MMT consists of the layered structure with octahedral and tetrahedral alumino-silicate sheet of T-O-T layers [56]. The MMT layered structure has to exfoliate via cation exchange capacity (CEC) [42], which might be helpful to incorporate CORMs commodity. The electro-kinetic and rheological characteristics of poly(ethylene glycol) into MMT interlayers favors the exfoliation [42]. The intercalation of polymer with MMT [57] and organically modified MMT drew our research team attention [58] to find MMT incorporating capability to apply it for CO-releasing strategic partnership.

**Figure 2:**
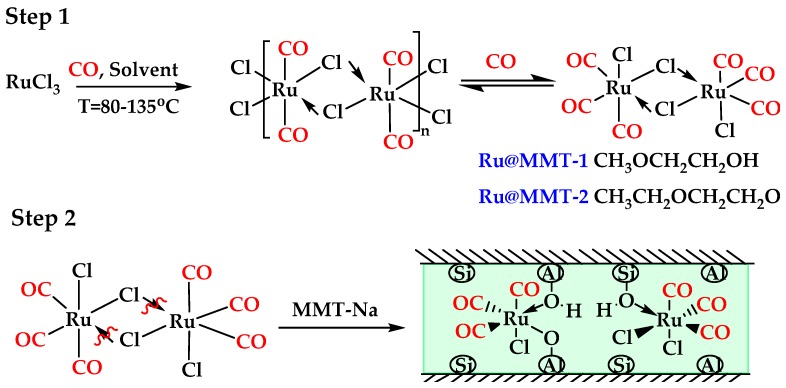
The development route for CORM’s precursor (Step 1); the CO administration strategy intercalates the CORM-2 inside the montmorillonite (MMT) layers (Step 2).

In the beginning, CO is induced in Ruthenium-organometallic complex through a carbonylation reduction reaction of RuCl_3_. RuCl_3_ is firstly transformed into [Ru(CO)_2_Cl]_2_ in a solvent of ethylene glycol monomethyl ether or ethylene glycol ether at a temperature of 80 °C. By proceeding the reaction mechanism, [Ru(CO)_2_Cl]_2_ change into [Ru(CO)_3_Cl_2_]_2_ (desire product) at a temperature of 135 °C. Meanwhile, HCl and H_2_O are generated as by-products. The whole process was carried out though a fast CO gas stream before the temperature reaches to 80 °C (Figure 2, Step 1). In the second and final step; the carbonyl complex of ruthenium complexes with the hydroxyl group in Na-MMT release the CO from clay MMT-RuCl_x_(CO)_y_ (Figure 2, Step 2). Different solvents have shown different degrees of exfoliation and adsorption that pursue the intercalation. Solvent is important for exfoliation to incorporate with different solvents. It possesses different characteristics according to the fabrication of CORMs. Furthermore, this layers stretching played a vital role for the ruthenium-loaded interlayer of MMT. The reaction mechanism can be classified into two different routes because the carbonylation reduction mechanism is independent and is a reversible reaction. Whereas the ruthenium carbonyl is easy to load inside the MMT layers after successfully fabricating it with the support of appropriate solvent.

After releasing the CO fragment, MMT layered structure has the ability to contain the transition metal residue inside the layers spacing [59]. As a result, we are able to restrict the toxicity of MCCs. Thus we claimed the safe releasing mechanism with biocompatibility. Moreover, porosity, microscopic level diffusion rate [60], swelling capability [61,62] and degree of penetration together with adsorption characteristic [63] facilitate the MMT for scaffolding. We are going to present the novel transport drug services montmorillonite that is already reported as a safe way of curing keeping in view all the aforementioned challenges and demanding features of prerequisite pro-drug.

## 2. Characterization Techniques

### 2.1. Infrared Spectroscopy (IR)

**Figure 3:**
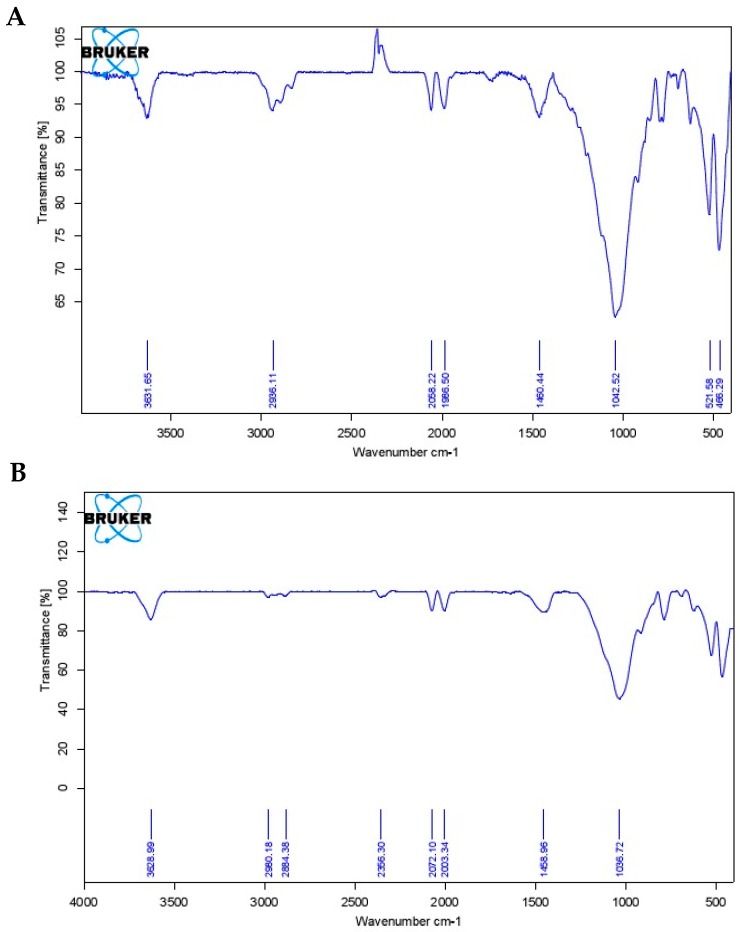
The Infrared (IR) spectrum of MMT-RuCl_x_(CO)_y_ complexes after carbonylation of montmorillonite with CORM-2 [Ru(CO)_3_Cl_2_]_2_ of Ru@MMT-1 (**A**) and Ru@MMT-2 (**B**).

The reaction mechanism is monitored through Infrared spectroscopy. When carbonylation is completed using ethylene glycol monomethyl ether as solvent (Ru@MMT-1), the ruthenium metal carbonyl coordination appears at 1986 and 2058 cm^−1^ absorption bands. Furthermore, the absorption band at 3631 cm^−1^ is caused by the stretching vibration of the hydroxyl group in the Al-OH and the vibration of the Si-O-Si skeleton has been found at 1042 cm^−1^ band (Figure 3A). When solvent is replaced with ethylene glycol ether (Ru@MMT-2) same observation has been monitored with slightly different absorption bands. The 2072 and 2003 cm^−1^ band referred to the ruthenium metal carbonyl coordination. The 3628 cm^−1^ band is caused by the stretching vibration of the hydroxyl group in the Al-OH, and the Si-O-Si skeleton vibration at 1036 cm^−1^ band as shown in IR spectra (Figure 3B).

### 2.2. Scanning Electron Microscopy (SEM)

**Figure 4:**
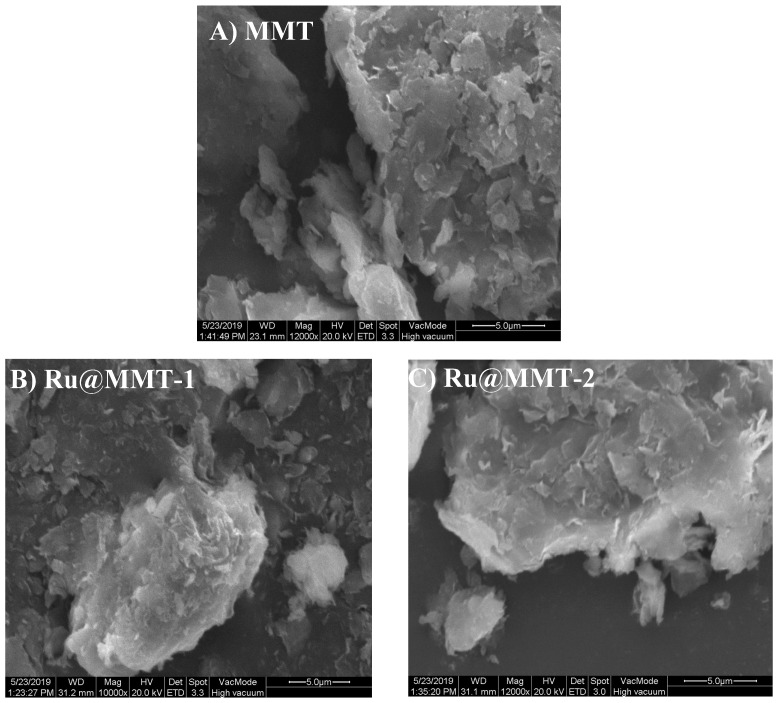
The Scanning electron microscopy (SEM) micrograph of Na-MMT (**A**), Ru@MMT-1 (**B**) and Ru@MMT-2 (**C**) at 5.0 μm provided the information about the layers formation.

SEM images of sodium montmorillonite after CORM-2 intercalation (Ru@MMT-1, Ru@MMT-2) are shown in Figure 4. It has been clearly identified that the montmorillonite layered structure has not been damaged. Since some particles were rearranged but did not lose their crystallinity as was in the foundation of the layered structure.

### 2.3. Transmission Electron Microscope (TEM)

**Figure 5:**
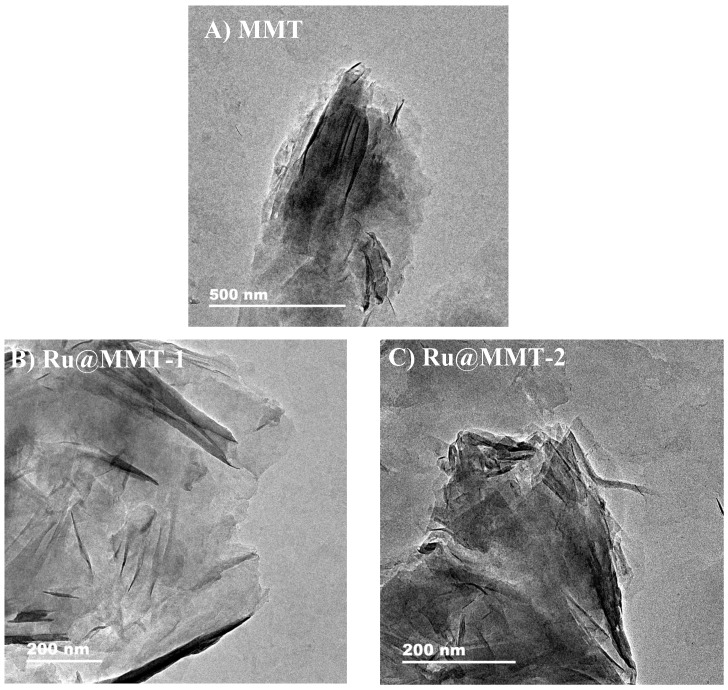
The Transmission electron microscope (TEM) of Na-MMT (**A**), Ru@MMT-1 (**B**) and Ru@MMT-2 (**C**) clarified their layered structures existence.

SEM provided the information about the montmorillonite layer structure confirming that it has not been destroyed. However, the interlayers’ interaction can be explained by performing the Transmission electron microscope (TEM) analysis. TEM provided the collection of data at microscopic level (crystal lattice) to analyze the structural transformation of MMT, surface properties and layers interaction with COMR-2 through different solvent. The characterization of natural montmorillonite explained by the particle morphology that could be specific to the different genesis along with wide spread of welted edges, might be helpful for obtaining lattice visualization. We found no significant contrast at 200 nm scale-bars among montmorillonite, Ru@MMT-1 and Ru@MMT-2 (Figure 5). Through TEM images we can conclude that flakes of MMT persist before and after synthesis. Simply we can say that the layered structure has not been transformed. On the other hand, we can be assuming that layered structure involved in cation-exchange at different octahedral and tetrahedral edges of MMT.

### 2.4. X-Ray Diffraction (X-ray)

**Figure 6:**
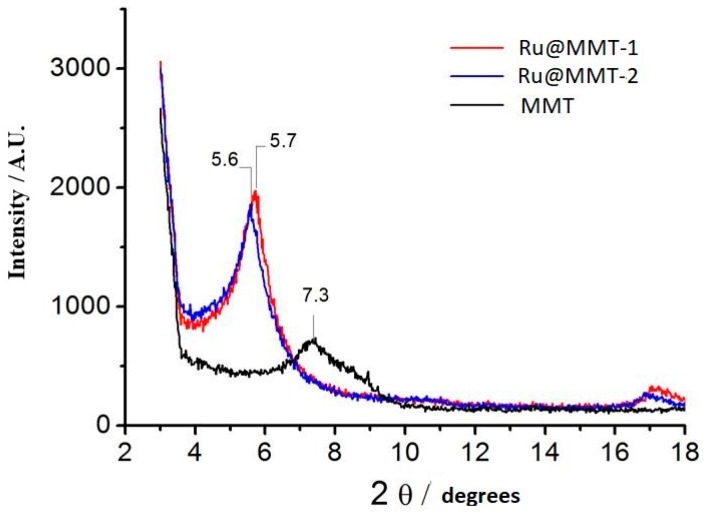
The X-ray diffraction (X-ray) observed the characteristic peaks of Ru@MMT-1 and Ru@MMT-2 at 5.7 and 5.6 degree as a consequence of exfoliation.

After the confirmation of layered structure, the layered exfoliation was investigated through X-ray diffraction; this exfoliation helps to understand the intercalation strategy. Using X-ray we found the diffraction of montmorillonite, Ru@MMT-1 and Ru@MMT-2; the characteristic peaks at 5.7 and 5.6 degree confirms the structure of material (Figure 6). The montmorillonite has been exfoliated and this layers exfoliation is helpful for the carbonylation and Ruthenium insertion.

### 2.5. Thermogravimetric Analysis (TGA)

**Figure 7:**
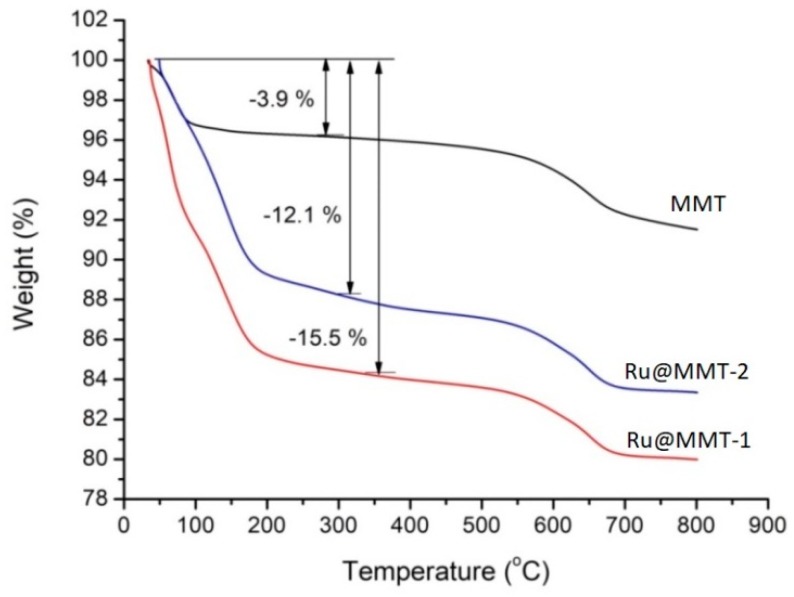
The thermogravimetric (TG) curve of MMT, Ru@MMT-1 and Ru@MMT-2 described their weight loss percentage upon temperature gradient.

The montmorillonite peeling layer was further characterized by thermogravimetric analysis (TGA) to observe the adsorbed quantity inside the layer. For MMT, the weight loss rate in the first step of weightlessness was 3.9% due to the loss of coordination water in the MMT layered structure. The TGA characterization of Ru@MMT-1 and Ru@MMT-2 showed a decrease in mass with increases in temperature, and the weight loss was greater at the initial stage due to solvent volatilization, loss of coordination water and cleavage of Ru-CO bond, and their corresponding weight loss rate were 15.5% and 12.1% respectively (Figure 7). After the temperature reached at 200 °C, the thermogravimetric curves of Ru@MMT-1 and Ru@MMT-2 were similar to the thermogravimetric curve of montmorillonite, indicated that the coordination bond on Ru@MMT-1 and Ru@MMT-2 were no longer cracked, meanwhile layered structure of montmorillonite itself prolonged. The TGA describes that the adsorbed content decreased under the temperature gradient and explains the temperature limit of the reaction. This figure helps to understand the temperature profile of Ru@MMT-1 and Ru@MMT-2 too. Eventually, it can be observed that solvent molecules have been adsorbed inside the layers of MMT, which is confirmed in corresponding to the XRD results.

### 2.6. Energy-Dispersive X-Ray Spectroscopy (EDX)

**Figure 8:**
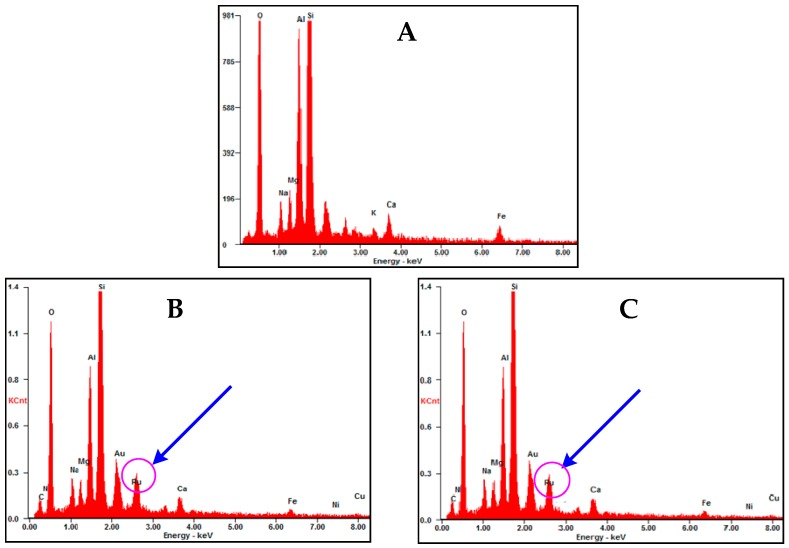
The Energy-dispersive X-ray spectroscopy (EDX) of pure Na-MMT (**A**) can be compared with Ru@MMT-1 (**B**) and Ru@MMT-2 (**C**), identified the existence of Ruthenium content inside the layers.

EDX spectroscopy was used to analyze the elemental composition of nanoparticles between the layered structures. EDX energy spectrum of both the products and the MMT was used to compare their elemental content (Figure 8). It is found that the metal content of Mg, Al, Si, Fe, and Pd have been reduced in final product as compared to the energy spectrum of pure MMT. The total amount of reduction (Ru@MMT-1: 8.21%, Ru@MMT-2: 14.57%) is greater than the mass percentage of Ruthenium metal content (Ru@MMT-1: 5.38%, Ru@MMT-2: 4.18%), which indicated that there must be cation exchange occurred between the MMT layers (Table 2). It can be assumed that Ru element entering the MMT layers and corresponding cations of these elements were also exchanged with H^+^ in the solvent. According to the EDX spectrum, Ruthenium content ratio present in Ru@MMT-1 and Ru@MMT-2 as 9:1 and 4:1 respectively (calculated from 0.3 mg of product).

### 2.7. Myoglobin Assay (Mb)

**Figure 9:**
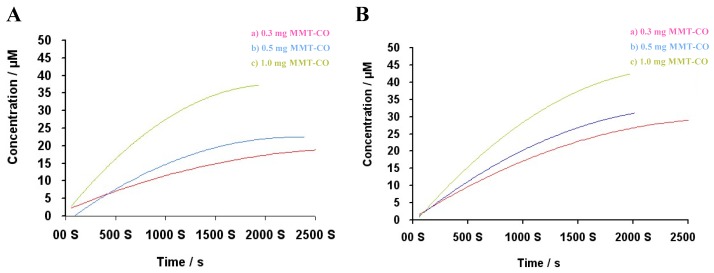
The kinetic release profile Ru@MMT-1 (**A**) and Ru@MMT-2 (**B**).

To test whether the [Ru(CO)_3_Cl_2_]_2_-functionalized montmorillonite might be appropriate for the liberating agent of carbon monoxide to the biological environment. CO released mechanism was studied through standard myoglobin assay [64]. For that prospective, horse skeletal myoglobin (Mb) (a phosphate-buffered aqueous solution PBS, pH~7.4) was freshly reduced deoxy-Mb (dMb) through excess sodium dithionite (Na_2_S_2_O_4_) under nitrogen environment, after metal-carbonyl [Ru(CO)_3_Cl_2_]_2_ -functionalized montmorillonite was added. When CO gas is passed into dMb solution, color of the solution turns red then the spectrum of the saturated Mb-CO at different concentration (0.3 mg **a**, 0.5 mg **b**, and 1 mg **c**) is tested as illustrated in kinetic release profile (Figure 9). Ru@MMT-1 has been observed to release the maximum amount of CO at 18.36 uM (**a**), 22.788 uM (**b**), and 37.612 uM (**c**) and their half-lives were recorded as 10 min, 12 min, and 11 min respectively (Figure 9A). We observed that as the concentration of Ru@MMT-1 was increased then amount of CO released also increased without too much affecting their half-lives. But, In Ru@MMT-2 half-lives became shorter and showed fastest release the CO molecules in 14 min (**a**), 12 min (**b**), and 1 min **(c)** (Figure 9B). Although, CO released quantity increased when the concentration was increased such as 30.170 uM (**a**), 31.064 uM (**b**), and 42.461 uM (**c**). In comparison, the half-life of CORM-3 is 3.6 min when anticipate with plasma [65], but Ru@MMT-1 and Ru@MMT-2 has a greater half-life, indicating that MMT has excellent sustained effect.

## 3. Discussion

IR spectrum shows the ruthenium metal carbonyl coordination at different CO absorption bands (1986 ~ 2058 cm^−1^). Coordination of carbonyl complexes compared with various other CORMs infrared spectroscopy data such as Sawhorse CORM (1942, 1972 and 2026 cm^−1^) [66] and [Mn(tpm(CO)_3_]^+^ (1941 and 2047 cm^−1^) [13]. This CO insertion was caused by MMT layers exfoliation. The MMT layered behavior was explained through XRD and TG analysis. The XRD diffraction showed the layers diffraction (Ø) deviated from 7.3° to 5.7° and 5.6°. This diffraction is associated with the basal spacing. It also confirmed the enlarged interlayer spacing of MMT. This layers stretching is also monitored from the temperature gradient profile of TG curves. At 200 °C, weight loss rate of Ru@MMT-1 and Ru@MMT-2 were 15.5% and 12.1% respectively. Strategically Ru@MMT-2 has more molecular weight of solvent than Ru@MMT-1, but weight loss of Ru@MMT-1 is greater than Ru@MMT-2, we can assume that Ru@MMT-1 adsorb more solvent than Ru@MMT-2. MMT exclusive feature of the adsorption is also described through the TG analysis. The layered structures formation is observed form the SEM and TEM images.

EDX spectrum analysis confirmed the Ruthenium presence in [Ru(CO)_3_Cl_2_]_2_ -functionalized montmorillonite. According to the EDX spectrum, the mass percentage (Wt%) of the ruthenium element contained in the two samples were 5.38% and 4.18% respectively (Table 3). In comparison with EDX spectrum data of pure Na-MMT, it was found that the amount of Ruthenium present in the two products, we consider 0.3 mg sample of each product. It can be seen that the ratio of ruthenium element to carbonyl group in the two samples is about 9:1 and 4:1, respectively.

N_1_ = 0.0003 ∗ 5.38% g/101.07g   mol^−1^ = 0.1597 umol.

N_2_ = 0.0003 ∗ 4.18% g/101.07g   mol^−1^ = 0.1240 umol.

Considering the release kinetic curve at two different concentrations of 0.3 mg sample product (Figure 9), it can be seen that the equilibrium concentrations of Mb-CO were 18 uM and 28 uM respectively. The volume of Mb-CO is 1000 uL. Thus, the amount of Mb-CO is 0.018 and 0.028 umol respectively. According to the relationship of *CO* + *Mb* → *Mb-CO*, it was calculated that the amounts of carbonyl groups contained in the two products were 0.018 umol and 0.028 umol respectively (back-calculation). As the concentration of Ru@MMT-1 increases, the released CO amount also increased, although their half-life was relatively close to each other. In the case of Ru@MMT-2, as the concentration increases, the released CO amount also increased. Thus, the releasing mechanism becomes faster and faster. Compared with CORM-3 half-life (3.6 min, we found that CO-MMT has a higher half-lives indicating that MMT has a better-sustained release effect.

The broad scope of Ru@MMT CO-releasing segment has many advantages over different established transport services. Materials such as graphene oxide sheet (GO nanosheet) and metal organic framework (MOFs) are unable to contain the toxic metal fragment and metal leaching dilemma, which becomes a challenge for therapeutic applications.

## 4. Materials and Synthesis Procedure

### 4.1. Materials

Montmorillonite, RuCl_3_, ethylene glycol monomethyl ether and ethylene glycol ether were purchased from Aldrich. All chemical used in this study were reagent grade. For carbonylation reduction mechanism, firstly inert environment of N_2_ gas was used and then switched to CO gas.

### 4.2. Characterization

#### 4.2.1. General Remarks

We used oven-dried Schlenk glassware for carrying out reactions mechanism under pure nitrogen atmosphere. The reaction vessels were covered by wrapping foil if required. Rotary evaporator RV 10 digital (Janke & Kunkel KG.IKA-Werk Company, Staufen Germany) was used to dry the product sample. Infra-red spectra were recorded on Bruker Tensor 27 IR spectrometer. Environmental scanning electron microscope (SEM) and energy-dispersive X-ray spectroscopy (EDX) were performed on a FEI Quanta 200 microscope equipped with an EDX spectroscopy at an accelerating voltage of 30 KeV. Transmission electron microscope (TEM) was performed at JEM-2100 (Japan Company, Tokyo, Japan). X-Ray diffraction (XRD) was monitored by Burker D8 Quest (Burker, Karlsruhe, Germany). Thermogravimetric analyses (TGA) were performed at Q1000DSC + LNCS + FACS Q600SDT (American TA Company, New Castle, USA). The UV-Visible spectra were recorded at Japan company U-3900/3900H with high-resolution concave diffraction and high-sensitivity photomultiplier tube detector (wavelength range is about 190~900 nm).

#### 4.2.2. Preparation Ru@MMT-1

Add 0.0588g of RuCl_3_ and 20 mL ethylene glycol monomethyl ether to a three-necked flask, one port of flask is attached with condenser to regulate the temperate mechanism and another one is connected with the gas connection (N_2_/CO cylinders) for continues supply of gas to the reaction vessel. Meanwhile, we placed it at the heating plate. After-that, supply of N_2_ gas was opened for environment change (ensure the inert environment) and switched to CO at moderate rate (2 bubbles/s). Then heating system was turned and confirming the condenser reflux circulation and activate the magnetic stirrer at the rate of 570 rpm. After the adjustment of the whole apparatus, the oil temperature was set 80 °C. When the temperature reached at exactly 80 °C, then the reaction was carried out for 35 min. At the end of that time lapse, we observed that the physical appearance of the reactant has been changed, color of the solution changed from brownish black to blood red, confirming the chemical changes. At last, 1.0190 g of sodium montmorillonite (Na-MMT) was added to continue the reaction for 30 more minutes, until the color of the solution turned into egg yellow. After observing the yellow appearance the heating was stopped and mixture was cooled down to room temperature. The product was transferred into a round bottom flask. The flask was placed into rotary evaporator for drying. We obtained a pale grayish green solid Ru@MMT-1 of 1.1201 g. Finally, the as-obtained sample was sealed and stored at low temperature.

#### 4.2.3. Preparation Ru@MMT-2

The same procedure was repeated with different solvent ethylene glycol diethyl ether for produces the Ru@MMT-2. After the adjustment of the whole apparatus, oil temperature was set 80 °C. When the temperature reached at 80 °C, the reaction was carried out for 35 min until color of the solution changed from brownish black to blood red, confirming the chemical changes. Then temperature was raised to 135 °C for 20 min until the solution turned into golden yellow, then the temperature was lowered to 75 °C. At last, 1.0190 g of sodium montmorillonite (Na-MMT) was added to continue the reaction for 20 min, until the color of the solution turned into egg yellow. After observing the yellow appearance the heating was stopped and mixture was cooled down to room temperature. The product was transferred into a round bottom flask. The flask was placed into rotary evaporator for drying. We obtained a light gray green solid Ru@MMT-2 of 1.0247 g. Finally, the as prepared sample was sealed and stored at low temperature.

### 4.3. Myoglobin Assay for CO Kinetics Profile

Myoglobin (Mb) method was used to determine the CO release kinetics of [Ru(CO)_3_Cl_2_]_2_ -functionalized montmorillonite. The release of carbon monoxide by CORM was monitored by ultraviolet (Q-band region) absorption spectroscopy of *deoxy-Mb*→*carbon-oxymyoglobin* (Mb-CO) changes. The liberated CO molecules from the nanocomposite, bind with Mb to form MbCO; and thus the concentration of Mb-CO was quantitatively determined by measuring the change in absorbance from 500 nm to 600 nm UV spectrums. 10 mg of horse skeletal muscle myoglobin (Mb) was dissolved in 5 mL volumetric flask of phosphate-buffered saline (PBS) (0.1 M, pH = 7.4) and located into quartz cuvette (*l* = 1 cm). Then UV spectroscopic background was established. For this purpose, add PBS buffer solution to the two cuvettes and record the UV spectrum from 500 nm to 600 nm for completing the background correction to ensure the baseline is zero, which was used as background spectrum for reference. After that, the solution has to degas through bubbling of dinitrogen. Since, the myoglobin was reduced to deoxy-Mb by the excess addition of 100 μL sodium dithionite Na_2_S_2_O_4_, then charge into same solvent with total volume 990 μL (0.1% saturation), and recorded the UV/Vis absorption spectrum of the reduced myoglobin (deoxy-Mb, dMb). When the sufficient amount of carbon monoxide gas was bubbled into the mixture (0.1% saturated solution), then color of the solution turns red. After that, the spectrum test of saturated Mb-CO has been carried out. 1000 uL (1 mL) of dMb solution was obtained and the nanocomposites (about 0.3 mg CORMats sample) were quantitatively uniformly dispersed in the transport solvent dimethylsulfoxide (DMSO) with the minimum amount. After mixing quickly, 5–6 drops of paraffin oil were added to prevent CO from spillage and deoxy-Mb from being oxidized again. Hence, the final concentration was maintained up to 1 mM of sodium dithionite (Na_2_S_2_O_4_) and 4 mM, 8 mM and 12 mM of prepared DMSO mother liquors along 20 μM, 40 μM and 60 μM of myoglobin. The cuvettes were placed in the UV spectrophotometer sample chamber and started recording the spectrum until no longer significantly changes in spectrum variation appeared. For measuring the CO-MMT kinetic release profile, the same procedure was repeated with two products Ru@MMT-1 and Ru@MMT-2 at different concentrations 0.3 mg, 0.5 mg, and 1 mg respectively. The same sample of the same concentration was treated with the same Mb solution.

## 5. Conclusions

Based on transition metal carbonyl compounds of carbon monoxide- releasing molecules in the hypertension, lung damage and organ allograft rejection interventional treatment such effect is obvious, has successfully replaced carbon monoxide gas fields of biomedical research to become the new hot spot. But the toxicity of such release molecule itself and the release of toxic metal residues limit its application as a therapeutic drug precursor. This paper for the first time presents the pharmaceutical clay montmorillonite and deliberated as new class of carbon monoxide-releasing materials (CORMats) by successfully incorporating with CORM-2 and exhibited the good bio-compatibility as well. MMT is the first biocompatible material to capture the CO for therapeutic release. MMT is already prescribed in so many drugs formulations. The main advantage of using MMT is to reduce the toxicity of MCCs. Firstly, the crystalline layer structure of MMT has been exfoliated using solvent penetration then cation exchanged capability favors the ion exchange between the MMT layers and CORM-2′s commodity either swapping or inserting. SEM and TEM images confirmed the layered structure. The Ruthenium metal carbonyl coordination has been verified by IR spectroscopy using absorption bands at 1986 cm^−1^, 2058cm^−1^ (Ru@MMT-1) and 2072 cm^−1^, 2003 cm^−1^ (Ru@MMT-2). The XRD characterized the layered exfoliation using diffraction parameter. EDX analysis assured the Ruthenium presence (Ru@MMT-1: 5.38%, Ru@MMT-2: 4.18%) and myoglobin assay acknowledged the CO liberation to biological system. The determination of CO sustained release shows that the CO releasing rate of prepared CO-MMT (maximum half-live ~ 14 min) has been much higher than CORM-3 (3.6 min) with an excellent sustained release effect. The heavy metal adsorption of montmorillonite could end up solving transition metal carbonyl compounds release problem of residual heavy metal ions; thus providing a treatment for carbon monoxide-releasing new material. The metal leaching and reduced the toxicity of MCCs have been well addressed in this article.

CO has therapeutic potential for Ulcerative Colitis disease and MMT has the ability to transport the CO analogous to diseased organs. Hence, the biocompatible pharmaceutical drug MMT has a potential for curing the murine colitis disorders. In the future, we will present the clinical study about Ulcerative Colitis in rats.

## Figures and Tables

**Table 1:** The different administrations of CO for therapeutic purpose.

Sr. No.	Strategic Advantages	CO	CORMs	CORMats (This Work)
1	Therapeutic ways	Direct insertion	Indirect insertion	Indirect insertion
2	Constructability	Not recommended	Possible	Possible
3	Controllability	Proper arrangement required	Possible	Possible
4	Administration capability	Proper arrangement (In-need hospital)	Itinerant	Itinerant
5	Loading capability	High	Low	Controllable
6	Specific equipment	Yes	No special requirement (Just orally intake)	No special requirement (Just orally intake)
7	Targeting tissue facility	Nearly impossible	Feasible, moderate Control	Feasible, more Control
8	Tissue selectivity	Not prefer	Prefer	Confident
9	Toxicity of MMCs	Not present	Difficult to control	Reduced toxicity
10	Tissue receptor	Impossible	Need special arrangement	Easy to modified

**Table 2:** The comparison of main element content is present in two products before and after degradation.

Mass Percentage [Wt%] ^1^	MMT ^2^	Before Degradation		After Degradation	
		Ru@MMT-1	Ru@MMT-2	Ru@MMT-1	Ru@MMT-2
Mg	2.26	1.81	1.41	1.99	1.87
Al	10.54	8.99	7.34	12.44	13.01
Si	34.40	29.72	25.76	40.15	38.04
Ru	0.00	5.38	4.18	3.81	2.71

^1^ EDAX ZAF Quantification Standardles, ^2^ montmorillonite before treated.

**Table 3:** The detail EDX spectrum data analysis of Ru@MMT-1 and Ru@MMT-2 compared with pure Na-MMT.

Elements ^1^	Montmorillonite ^2^		Ru@MMT-1		Ru@MMT-2	
	Wt [%]	At [%]	Wt [%]	At [%]	Wt [%]	At [%]
O K	43.20	58.07	35.55	55.33	33.20	46.29
Na K	02.41	02.25	02.97	03.21	02.40	02.33
Mg K	02.26	02.00	01.81	01.85	01.41	01.30
Al K	10.54	08.40	08.99	08.30	07.34	06.07
Si K	34.40	26.34	29.72	26.35	25.76	20.46
Pd L	01.35	00.27	00.63	00.15	00.15	00.03
Ca K	01.98	01.06	02.06	01.28	01.52	00.85
Fe K	03.15	01.21	01.53	00.68	01.27	00.51
Ru L	0.00	0.00	05.38	01.33	04.18	00.92

^1^ EDAX ZAF Quantification Standardles, ^2^ montmorillonite before treatment.
